# Cyanoacrylate glue for closure of proximal enterocutaneous fistula: a case report

**DOI:** 10.1093/jscr/rjab165

**Published:** 2021-05-05

**Authors:** Ahlam Hamed Alharbi, Abdularahman M Alotaibi

**Affiliations:** 1 Department of Surgery, Faculty of Medicine in Rabigh, King Abdulaziz University, Jeddah, Saudi Arabia; 2 Department of Surgery, Dr. Soliman Fakeeh Hospital, Faculty of Medicine in Jeddah, Jeddah University, Jeddah, Saudi Arabia

## Abstract

Enterocutaneous fistula (ECF) is a distressing complication. Commonly, it follows abdominal operations that require extensive adhesiolysis. Its management is challenging, burdening health systems. Complete healing can take several weeks. Several modalities have been described, with varying success rates. A 48-year-old male underwent a trauma laparotomy, with resection of a segment of the proximal bowel and anastomosis. He experienced an anastomosis leak, wound infection and ECF and was managed conservatively for 5 weeks with parenteral nutrition and bowel rest. He was then referred to us and treated with approximation sutures and cyanoacrylate adhesive. His wound was closed with a subcutaneous drain. He experienced complete healing of the fistula and wound after undergoing a minimally invasive approach using sutures and a cyanoacrylate sealant. Cyanoacrylate glue is a safe initial non-invasive treatment of low-output ECF. It can be selected over approximation sutures to ensure sealing of the tract before surgery.

## INTRODUCTION

Enterocutaneous fistula (ECF) is a connection between the skin and gastrointestinal tract [1] and a complication that requires costly and lengthy hospitalizations. The most common cause is iatrogenic, accounting for 75–85% of cases [2], and primarily occurs post-surgery with extensive adhesions release. Other causes include trauma, malignancy, infection, Crohn disease, bowel leak and radiotherapy. ECF is classified, based on anatomical origin into type 1 (abdominal, oesophageal, gastroduodenal), type 2 (small bowel), type 3 (large bowel) and type 4 (enteroatomospheric) [1]. Its management includes non-operative intervention for at least 5 weeks and operative intervention if the former fails. The aim of this paper is to discuss the effectiveness of a cyanoacrylate sealant in promoting closure of the ECF and review relevant articles.

## CASE REPORT

A 48-year-old male was referred to us from another facility after being treated for injuries due to a motor vehicle accident. Based on the operative report, he suffered from a severe mesenteric vascular injury with a 65-cm ischemic proximal small bowel segment, undergoing resection and anastomosis with primary abdominal closure. After recovery and discharge from the ICU, he developed a wound infection, bowel content discharge and dehiscence in the upper area of the wound, which was managed by frequent wet-to-dry dressing, TPN and low residual diet, begun as part of the management of the anastomosis leak. However, the wound remained open, and discharge persisted after 5 weeks; thus, he was referred to our facility for further evaluation and management.

**Figure 1:**
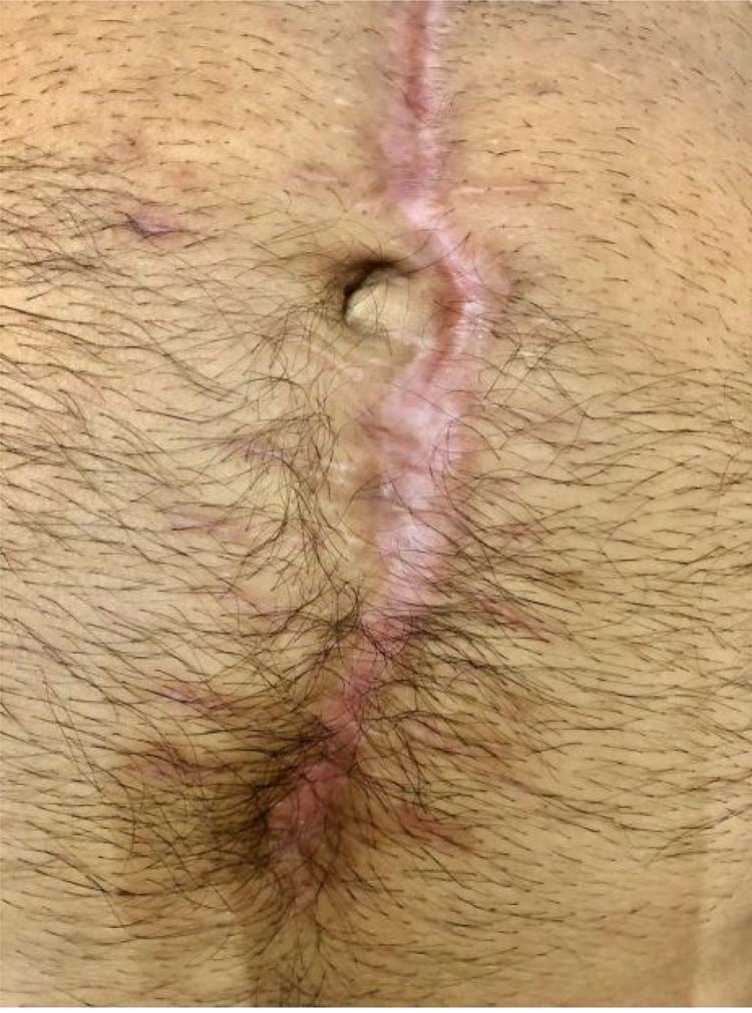
Pre-intervention.

**Figure 2:**
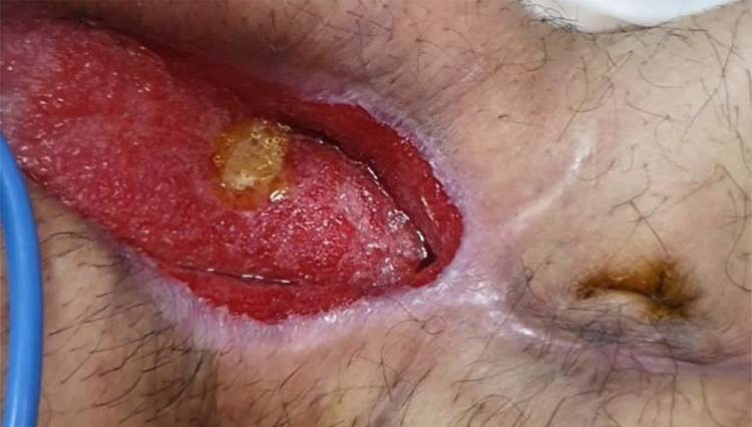
Post-closure.

On clinical examination, good granulation tissue was seen at the wound site, with greenish discharge, prompting a clinical diagnosis of proximal ECF (Fig. 1). The swap of the wound showed no contamination. The CT scan revealed gapping of the anterior abdominal wall with an underling small bowel but no contrast extravasation. The patient tolerated oral nutrition, with improved nutritional status. He was taken to the operating room, where a suture was used to approximate the external orifice of the fistula, with cyanoacrylate sealant (Glubran® 2, GEM Italy) applied to maintain the closure; consequently, the wound was closed after creating a flap of skin and subcutaneous tissue. A small drain with no suction was inserted under the skin. The patient was discharged on the second post-operative day. At the follow-up, the drain was removed after 7 days and the wound was clean, and the sutures were removed after 3 weeks. After 4 months, the healing was maintained, with no evidence of fistula or infection (Fig. 2).

## DISCUSSION

The use of tissue sealants such as fibrin glue and cyanoacrylate glue for the treatment of ECF began in the 1990s [3]. Reports on cyanoacrylate sealant have shown clear results in promoting healing of fistulae and decreasing the time of closure, with low output (<500 mL) and for proximally originating fistulae. Cyanoacrylate-based glue (Glubran 2) is a synthetic biodegradable material (n-butyl-2-cyanoacrylate and methacryl-oxysulpholane monomers) with high tensile strength; thus, it has high adhesive and hemostatic properties. It polymerizes once it makes contact with tissues and then creates an efficient antiseptic barrier that prevents the penetration of bacteria and infection. It has no direct adverse effects and minimizes the risk of transmitted infections (prions) that are associated with glue from animal sources (e.g. bovine). Furthermore, it is preferred over other materials, because it can be visualized by fluoroscopy when it is mixed with Lipidol [3–5].

ECF is managed conservatively with adequate nutritional support to maintain electrolytes and fluid balance, control the source of sepsis, protect the skin and lessen the output of the fistula using octreotide, with delineation of the tract anatomy. This preliminary treatment can last for 5–6 weeks before further intervention [6], with spontaneous closure in 15–71% of cases [2]. There are several factors in the rate of closure, such as anatomical origin, tract length, defect size and output [6]. Furthermore, the mortality rate can reach 37% [2]. For these reasons, intervention is indicated if the fistula does not close. Various approaches have been tested, such as endoscopic clipping, Gelfoam embolization, suture, plug and acellular dermal patch.

**Table 1:** Review of some studies and reported cases of ECF with a brief description of methods of treatment and healing rates.

Study (author), year	Number of cases	Type of treatment	Success rate
			
Sarfeh *et al.* 1992^10^	9 fistulae 6 patients (2 had 2)	Extraperitoneal closure with skin-graft coverage	5/9 healed 4/6 Recurrence in 2 patients requiring laparotomy and suture
Hwang and Chen *et al.* 1996^2^	6	Fibrin glue	Healed within 4 days
Girard *et al.* 2002^10^	Case report	Acellular dermal matrix with fibrin glue	Output stopped completely
Lisle *et al.* 2006^2^	Three cases of ECF	Radiologically guided embolization with Gelfoam	100% total occlusion in the 2-year follow-up
Ramón Rábago 2006^2^	30 patients with gastrointestinal fistulae (21 external)	Endoscopic injection of 4–8 mL of reconstituted fibrin glue (Tissucol® 2.0) on a weekly basis	Complete sealing of fistulae in 75%
Jamshidi *et al.* 2007^10^	7 fistulae (6 years review)	Application of biological dressings	43% achieved fistula closure solely with biological dressings
Lippert *et al.* 2010^6^	52 fistulae 7 in the small intestine	Endoscopic treatment, including fibrin glue (Tissucol Duo S®, Baxter, Unterschleissheim, Germany).	55.7% cured (combined endoscopic treatment) 36.5% (5/7) cured with fibrin glue as sole endoscopic option
Avalos-González *et al.* 2010^2^	Nonrandomized prospective case–control study 23 patients in the study group, 11 of whom had small bowel fistulae	Direct percutaneous application of fibrin glue	Closure-time for the study group was 12.5 ± 14.2 d and 32.5 ± 17.9 d for the control group
Wu *et al.* 2014^2^	Nonrandomized cohort study, 75 patients with low-output ECF	PRFG	77% success rate Median time of fistula closure was 7 vs. 23 days in control group. PRFG healed more fistulas within the first 28 days in 77 vs. 57%.
López *et al.* 2014^5^	Systematic review, 14 studies	Cyanoacrylate embolization	Ranged from 57 to 100% among studies
Araujo-Míguez *et al.* 2015^8^	Case report	Endoscopic treatment with biological fibrin glue (Tissucol Duo; Baxter).	Complete closure
Mauri *et al.* 2016^4^	18	Injection of (Glubran 2) cyanoacrylic glue and ethiodized oil mixture at the site of the fistula	89%
Musa *et al.* 2017^7^	Case report	Percutaneous injection of cyanoacrylic sealant	Complete closure
Hsu *et al.* 2017^6^	Case report	Hypertonic saline injection within the mucosa and use of fibrin glue as an adhesive	Complete closure
Nasralla *et al.* 2019^9^	Case report	Percutaneous embolization using cyanoacrylate glue/ethiodized oil mixture	Complete closure

Wu *et al.* treated 75 patients with platelet-rich fibrin glue (PRFG) and observed a lower median time of closure and more healed fistulae in the treatment versus control group, who was treated conservatively. The ideal time for administration of glue for post-operative fistula has been suggested to be 14 days post-stabilization of the fistula to promote healing and decrease the length of hospital stay [2], in contrast the timing of our application, which was affected by the referral time. Mauri performed percutaneous injection of cyanoacrylic glue to treat non-healing fistulae, noting a cure rate of 89% [4]. Others have described cases of post-surgical ECF that were treated successfully with an injection of cyanoacrylic sealant percutaneously, endoscopically or under radiological guidance, with good outcomes [7–9]. Multiple sessions of cyanoacrylate-based glue application might be needed to achieve complete closure in certain cases.

A summary of articles is presented in Table 1.

## CONCLUSION

Cyanoacrylate-base sealant is a safe and feasible non-invasive option for initial treatment of low-output ECF. It can be used over approximation sutures to ensure sealing of the fistula tract before advancement to more destructive surgery.

## CONFLICT OF INTEREST STATEMENT

None declared.
